# Mechanistic studies of the solvolysis of alkanesulfonyl and arenesulfonyl halides

**DOI:** 10.3762/bjoc.18.13

**Published:** 2022-01-17

**Authors:** Malcolm J D’Souza, Dennis N Kevill

**Affiliations:** 1 Delaware Environmental Institute, University of Delaware, 221 Academy Street, Newark, Delaware 19716-5804, USA; 2 Department of Chemistry and Biochemistry, Northern Illinois University, DeKalb, Illinois 60115-2862, USA

**Keywords:** correlation, Grunwald–Winstein equation, Hammett equation, mechanism, solvolysis, sulfonyl halides

## Abstract

There have been several studies on the solvolysis mechanisms for alkanesulfonyl chlorides (RSO_2_Cl) and arenesulfonyl chlorides (ArSO_2_Cl). The earlier of these studies were reviewed a little over thirty years ago by Gordon, Maskill and Ruasse (*Chem. Soc. Rev.* **1989**, *18*, 123–151) in a contribution entitled “Sulfonyl Transfer Reactions”. The present review will emphasize more recent contributions and, in particular, the application of the extended Grunwald–Winstein equation and kinetic solvent isotope effects to the solvolysis reactions. There is also an appreciable number of reports concerning the corresponding anhydrides with the chloride leaving group replaced by the appropriate sulfonate leaving group, concerning sulfamoyl chlorides (ZZ'NSO_2_Cl) with Z and Z' being alkyl or aryl and concerning the solvolysis of chlorosulfate esters (alkoxy- or aryloxysulfonyl chlorides), with the structures ROSO_2_Cl or ArOSO_2_Cl. The solvolyses of these additional types of sulfur(VI) substrates will be the topics of a future review.

## Review

### 1. Introduction to solvolyses of sulfonyl halides

In medicinal chemistry, sulfonyl chlorides are good building blocks to intermediates and complex scaffolds of sulfonamides. Gordon, Maskill, and Ruasse [1] have reviewed “Sulfonyl Transfer Reactions” in a broader sense than the coverage of the present contributions. In particular, an appreciable amount of the coverage in the earlier review involves nucleophilic catalysis of reactions carried out under solvolytic conditions, such that the species actually undergoing the solvolysis was an intermediate along the reaction pathway. This important aspect [2–3] will not be emphasized in the present review, where we will concentrate on reactions believed to involve a direct interaction with the substrate, or with a species (such as a cation) directly formed from the substrate.

Sulfonyl chlorides were included in several early considerations of the reaction rates, for processes involving organic substrates, as a function of solvent composition. In a very early 1927 study [4] of the solvolyses of benzenesulfonyl chloride (C_6_H_5_SO_2_Cl) and its *p*-methyl and *m*-nitro derivatives, solvolyzing in 50% acetone/50% water (v/v) at 25.0 °C, it was found that the differences in specific rates were quite small, at 0.0106 min^−1^ for the *p*-methyl derivative, 0.0146 min^−1^ for the parent compound, and 0.044 min^−1^ for the *m*-NO_2_ derivative. A change of solvent to 47.5% ethanol/52.5% water led to very similar behavior with the corresponding specific rates being 0.0287, 0.0313, and 0.058 min^−1^. In modern terminology, this would suggest a close to synchronous bond-making and bond-breaking, with a small negative charge development on the sulfur at the transition state, for the solvolytic process.

Subsequent to this 1927 study, Hammett reported [5–6] in 1937, the development of the Hammett equation, involving the consideration of a scale logarithmically related to the measured acidities for the parent benzoic acid and for a series of *para* and *meta*-substituted derivatives for a solution in water at 25.0 °C. In this initial study, *ortho*-substituents were avoided due to the possibility of steric interactions superimposed on the inductive ones.

The Hammett substituent values obtained can be applied to the solvolyses and other reactions of substrates varying only in the aromatic ring substituent that they carry, under otherwise identical conditions as regards solvent and temperature. Frequently, as in the reactions to be reviewed in this subsection of the review, the study is of solvolysis mechanisms and the reactions involve the solvolyses of a series of *para* and *meta*-ring-substituted substrates. Applying the Hammett equation to the rate measurements of Berger and Oliver [4], we arrive, in 50% water/50% acetone (v/v) at a reaction constant ρ value of +0.82 for the *para*-methyl derivative and of +0.71 for the *meta*-nitro derivative. and, for the study in 47.5% ethanol/52.5% water the corresponding values are +0.22 and +0.38, respectively. These small reaction constant ρ values depend only on the type of reaction but not on the substituent used, and are consistent with the proposed bimolecular character with a considerable balance between bond formation and bond breaking.

Hedlund [7] studied the rates of solvolysis for several alkanesulfonyl chlorides in 100% water. He found lower rates of solvolysis than those for the previously studied [4] arenesulfonyl chlorides. Böhme and Schurhoff [8] similarly found that the solvolyses, in several homogeneous mixtures of an ether and water, were relatively slow. They found that the overall reaction rate could be considerably increased by the addition of reasonably powerful anionic nucleophiles. Indeed, Swain and Scott showed [9] that in 50% acetone/50% water at 0.5 °C, a hydroxide ion was about 10^6^ times more reactive than a water molecule towards benzenesulfonyl chloride. Also, for the same solvent, at 25.0 °C, they found [10] that the fluoride, with a considerably stronger carbon–halogen bond, reacted at least four orders of magnitude slower than the chloride as regards the solvolytic replacement of the halogen present in the benzenesulfonyl halide. For the reactions of the fluoride in 20% dioxane at 91 °C, the *m*-nitro derivative was shown to be subject to a strong nucleophilic catalysis by acetate ion [11]. Hall [12] extended the studies of the hydrolyses of sulfonyl chlorides to sulfamoyl chlorides (ZZ'NSO_2_Cl, with Z and Z' each either alkyl or aryl) and to alkoxy (or aryloxy) sulfonyl chlorides ROSO_2_Cl or ArOSO_2_Cl, also named as alkyl (or aryl) chlorosulfates. These two classes of substrates will be considered in a subsequent review which will expand the coverage of the present one. It might be mentioned that Hall’s claim of an S_N_1 pathway for some of the solvolyses of dimethylsulfamoyl chloride has been rendered unlikely by more recent studies.

Tommila and Hirsjärvi [13] found that, in water as solvent, both electron-supplying and electron-withdrawing substituents decreased the rate of the hydrolysis of benzenesulfonyl chloride. They proposed, consistent with the claim by Hall [12], that with electron-supplying substituents the reaction proceeded, in part, by an ionization (S_N_1) mechanism. Similarly, Vizgert [14] studied the hydrolyses of the parent benzenesulfonyl chloride and a series of substituted derivatives in 70% dioxane/30% water. A modest increase in rate was observed for the 2,4-dimethyl derivative which then increased dramatically on going to the 2,4,6-trimethyl derivative. It was proposed that these observations constituted good evidence for the incursion of an S_N_1 pathway. A subsequent study by Vizgert and Savchuk [15] concentrated on the effect of gradually increasing the polarity of the solvent on going from 70% dioxane to 100% H_2_O. It was proposed that the rate changes observed as the water content increased were consistent with an S_N_2 reaction becoming a mixed S_N_2–S_N_1 situation and then, eventually, an essentially S_N_1 pathway was followed. It was concluded that nucleophilic attack at sulfur has many of the characteristics of attack at carbon, including the interplay of S_N_1 and S_N_2 pathways for attack at a saturated carbon atom being carried over to attack at sulfur(VI). From the experiments reported by Vizgert and Savchuk [15], we will choose just one of the many possibilities for an application of the Hammett equation [5–6]. Applying to the rate data in 30% dioxane/70% water at 20 °C, we can, for the parent benzenesulfonyl chloride and for the *p*-methyl and *p*-nitro derivatives, abstract the specific rate data from Table 1 of the publication. The Hammett treatment using appropriate substituent constants leads to a Hammett ρ value of +0.35 with the *p*-methyl group present and to a very similar value of +0.37 in the presence of a *p*-nitro group. Again, these low ρ values are consistent with a transition state with very similar amounts of bond-breaking and bond-making, such that only a small amount of charge develops at the α-carbon.

Over an eleven-year period (1961–1971), Hambly and co-workers, published a series of eight papers (Solvolysis of Sulfonyl Halides, Parts I through VIII) [16–23]. In the initial study [16–17] of the solvolysis of the parent benzenesulfonyl chloride and the *p*-methyl, *p*-bromo-, and *p*-nitro derivatives in aqueous dioxane, aqueous acetone, methanol/acetone, and ethanol/acetone, the *p*-NO_2_ (strongest electron-withdrawing influence) was always reacting faster than the parent and the other two derivatives and the overall behavior in a Hammett equation treatment indicated that all were reacting by an S_N_2 pathway. The plots all deviated modestly from linearity but overall an S_N_2 pathway with some degree of variation between the structures at the transition state was a logical assessment of the mechanistic situation. In a following publication [20], the solvolytic behaviors of methane- and ethanesulfonyl chlorides in the aqueous acetone and aqueous dioxane mixtures were reported. The evidence was again considered to be in favor of an S_N_2 pathway and the S_N_1 pathway, previously proposed [13–15] for reactions of sulfonyl chlorides in aqueous dioxane, was not supported by the Foon and Hambly data.

When the specific rates of solvolysis of ethanesulfonyl bromide and chloride, in aqueous dioxane at 25.0 °C, are compared as regards their variation with solvent composition [19], it is found that *k*_Br_/*k*_Cl_ values of modest magnitude fall from 10.9 for a mole fraction of water of 0.205 to a value of 2.8 at a mole fraction of water of 0.990. If the reactions were S_N_1 in character with a larger proportion of water in the water/dioxane mixtures, the values would be expected to decrease appreciably because of the stronger solvation by water of a developing chloride ion than of a developing bromide ion [24]. For S_N_1 reactions, *k*_Br_/*k*_Cl_ ratios are usually in the region of 40 [24] and much smaller values, similar to the ones of this study, are considered to reflect S_N_2 character.

Solvent isotope effect values (*k*_H2O_/*k*_D2O_) for the solvolyses of methanesulfonyl chloride have been determined [20]. The value for the ratio decreased slightly with increase in temperature, from a value of 1.7 at 0 °C to 1.5 at 50 °C. When the H_2_O and D_2_O are diluted with gradually increasing amounts of dioxane, there are only very small changes in the solvent isotope effect ratio. The individual specific rates of solvolysis increase by a factor of 2.3 when the pressure is raised to 2000 atmospheres but the solvent isotope effect ratio is essentially unchanged [21].

The parent benzenesulfonyl chloride and a series of monosubstituted derivatives (*p*-nitro, *m*-nitro, *p*-bromo, *p*-methoxy, and *p*-methyl) have had their specific rates of hydrolysis measured in aqueous dioxane [22]. The observations concerning the specific rates were consistent with an S_N_2 pathway for the displacement of the chloride ion from the sulfur. However, for the trisubstituted 2,4,6-trimethylbenzenesulfonyl chloride, the S_N_2 mechanism does not give a simple rationale for the high specific rate of solvolysis observed and a partial changeover to an S_N_1 pathway was proposed. Vizgert [14–15] had previously suggested that the favored pathway for the hydrolysis of this substrate was by an S_N_1 ionization mechanism [25]. This proposal was based on the lack of acceleration in aqueous dioxane when hydroxide ion was added. As the polarity of the medium is increased, so also is the solvent ionizing power (*Y*) and the ionization pathway could become of increasing importance and eventually dominant.

Forbes and Maskill, first in a communication [26] and then later in a full-length paper with contributions from their co-workers [27], realized that if the unimolecular reaction was occurring in aqueous dioxane solvents, then it should become even more dominant when the dioxane was replaced by 2,2,2-trifluoroethanol (TFE). Charge density distributions for TFE show a component with decreased electron density at the oxygen, which is relayed to give increased acidity for the hydrogen of the OH group and increased electrophilicity (solvent ionizing power) for a solvent in which it is a major component. Maskill and co-workers [26–27] investigated the solvolyses of 2,4-dimethoxybenzenesulfonyl chloride and of the 2,4,6-trimethylbenzenesulfonyl chloride in 50% TFE/50% water (50% TFE) and in the highly ionizing 97% TFE. They found no evidence for an unusually rapid reaction in these solvents and very negative entropies of activation (consistent with a bimolecular process). If in these highly ionizing solvents no changeover from an S_N_2 to an S_N_1 process is observed, then it certainly would not be expected under the much milder conditions of aqueous dioxane solvents [26–27].

It would, in turn, be worthwhile to attempt to repeat these experiments with the TFE replaced by the even more electrophilic 1,1,1,3,3,3-hexafluoro-2-propanol (HFIP) component. The 97% TFE has a *Y*_Cl_ value (for interaction at chlorine) of 2.83, which increases to 5.08 on going to 97% HFIP [25], corresponding to a considerable increase in its ability to interact with, and assist in the removal of (as chloride ion), a chlorine attached to a carbon.

Robertson and co-workers devised a system for a very accurate determination of rates of reaction by following changes in conductivity, with very precise temperature control. In this way, it was possible to observe systematic deviations from linearity in Arrhenius plots, which with conventional kinetic studies would be masked by a larger random scatter within the different determinations. A precise detailed treatment allowed the “second-order” heat capacities of activation (∆*C_p_*^#^) to be determined for, among many other substrates, methane and benzenesulfonyl chlorides [28]. The magnitude of the ∆*C_p_*^#^ was initially considered to be the most sensitive indicator of the extent of solvent reorganization during the substitution process. Similarly, a very accurate determination of the kinetic solvent isotope effect (*k*_sie_) for solvolysis in H_2_O or D_2_O based on very accurate specific rate measurements was possible. The *k*_sie_ for the hydrolysis of alkyl chlorides is usually in the range of 1.20 to 1.25 which, at 20 °C, increases to values of 1.568 ± 0.006 and 1.564 ± 0.006 for methanesulfonyl chloride and benzenesulfonyl chloride [28]. This was considered to indicate more bond breaking at the transition state for the departure of the chloride ion in the hydrolyses of the sulfonyl chlorides.

The sulfonyl halides are not ideal substrates for studies of this type since they range from poorly soluble to almost insoluble in water. The parent compounds distinguished strongly between reactions with a water molecule and added hydroxide ion. The selectivity ratio [10], *k*_OH_−/*k*_H2O_ with benzenesulfonyl chloride as the substrate having a value of 7 × 10^8^, considerably larger than the value of 10^3^ for primary alkyl halides [4,16–17] and consistent with an S_N_2 pathway. Introduction of methyl groups onto the aromatic ring considerably reduced the selectivity ratio, with specific rate values [14] of 1.66 × 10^−4^ s^−1^ in 100% H_2_O increasing only to 1.73 × 10^−4^ s^−1^ in the presence of 0.004 M potassium hydroxide. The only small difference between the specific rates of reaction with solvent water and the overall value with added hydroxide ion suggested [14], but does not demand, a unimolecular S_N_1 pathway [28]. Overall, it was concluded [28] that the hydrolyses of both the methane- and benzenesulfonyl chlorides usually involve an S_N_2 pathway.

In a subsequent paper [29], for the solvolyses of benzenesulfonyl chloride and its *p*-MeO, *p*-Me, *p*-Br, and *p*-NO_2_ derivatives specific rates of hydrolysis were measured in the 0 °C to 25 °C range. The thermodynamic parameters, ∆*G*^#^, ∆*H*^#^, and ∆*S*^#^ and the above mentioned second-order parameter ∆*C_p_*^#^ were derived. The reactions were believed to be S_N_2 in character and a trigonal bipyramidal transition state was proposed. Electron withdrawal led to higher values of ∆*H*^#^ and less negative values of ∆*S*^#^. The ∆*C_p_*^#^ values correlated very well against ∆*S*^#^ values, except for an unusually low value for the *p*-MeO derivative. Arguments to explain this [29] were presented at length but do not seem to be totally convincing.

**Scheme 1:**
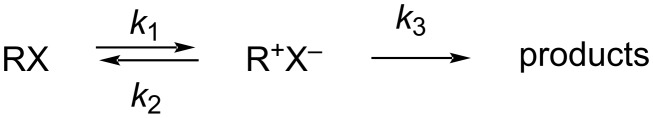
Organic reactions where the breaking of a C–X bond involves the formation of a high energy ion-pair intermediate.

In a third paper [30], kinetic solvent isotope effects (*k*_sie_ values) were reported as *k*_H2O_/*k*_D2O_ for the same group of benzenesulfonyl chlorides as reported on in part II [29]. Differences are found, as one might predict, in the nucleophilic processes for attack by water on sulfur rather than on carbon, indicating differences in the detailed bimolecular processes. In the paper, a scheme is presented which involves formation of an ion pair which can then either return to reactant or go on to products (Scheme 1).

The introduction of the ion-pair return (*k*_2_) step might seem rather trivial but it has profound effects as regards interpretation of the thermodynamic parameters. The general assumption until this expansion of the pathway was that the specific rate (*k*) was either about the same throughout the series of substituted compounds or it varies linearly with the σ values of the substituents. More recently, several groups have zeroed in as regards the incorporation of Scheme 1 into the development of a new model for solvolytic reactions and it has been shown conclusively, as Robertson gracefully acknowledged, in his participation as a co-author of a chapter in “Progress in Physical Organic Chemistry” [31], that this equilibrium needs to be incorporated into treatments based on the magnitudes of the heat capacities of activation (∆*C_p_*^#^). The need to incorporate the return from an ion pair to a covalent molecule, makes what was already a rather controversial area of reaction kinetics even more complicated and controversial. The reader wanting more detail is referred to the above mentioned comprehensive review entitled “Solvolysis Revisited” [31].

### 2. Application of simple and extended forms of the Grunwald–Winstein equation

For a rigid explanation of how the substitution reactions of sulfonyl chlorides proceed during solvolysis, a detailed picture of the influence of the solvent as one proceeds from reactants to products, such as one developed based on the procedures outlined above [31] would be required. However, if one is willing to settle for a classification of the mechanism as unimolecular or bimolecular accompanied by an approximate measure of the extents of bond-making and bond-breaking at the sulfur atom involved at the transition state, one can use a linear free energy relationship (LFER) approach [32].

Probably the best known LFER is the Hammett equation which presents a way of correlating the behavior of reactants with a substituent present in an aromatic ring with the effect of that substituent on the acidity of benzoic acids in water at 25 °C. As one would expect as once moves away from the standard reaction, the goodness of fit of the data using the Hammett substituent constants will be decreased. For example, for phenols which have a direct conjugation of a developing anion with an appropriate substituent, adjusted σ values are available for these substituents.

The above is presented to give some background to the approach that can be used to give one type of information that can be useful in studies of solvolysis reactions in which the solvent for the substrate is also the reactant. The substrates that are being correlated are those which can give substitution products, in many (but not all) cases accompanied by elimination products. The original equation was developed by Grunwald and Winstein in 1948 [33] and, on the occasion of its sixtieth birthday, the authors of the present review published a brief account of the development and uses to commemorate the occasion [34].

Accordingly, we will be somewhat brief in the introduction to this treatment before discussing its application to a study of solvolyses taking place at the sulfur atom of sulfonyl chlorides. The original scale of solvent ionizing power (*Y*) values was based on a study of the solvolyses of *tert*-butyl chloride in a variety of aqueous/organic solvents at 25.0 °C and the LFER was expressed as in Equation 1, where *k*_o_ is the specific rate (first-order rate coefficient) for solvolyses in the arbitrarily chosen standard solvent of 80% ethanol and 20% water (by volume) and *k* is the corresponding specific rate in some other pure or mixed solvent. The sensitivity value *m* is set at unity for the standard solvolysis and then for some other solvent the solvent ionizing power (*Y*) will be given by log (*k*/*k*_o_). Subsequently, with the ready availability of cage compounds (1-adamantyl and 2-adamantyl with a leaving group X), the moderate to weak nucleophilic participation with *tert*-butyl derivatives [35] can be avoided. The more reactive 3° compound is used to set up scales of *Y* values for the relatively poor leaving groups and the less reactive 2° compound is used for the relatively good leaving groups [25].


[1] $$\log(k/k_{o})=mY$$

**Scheme 2:**
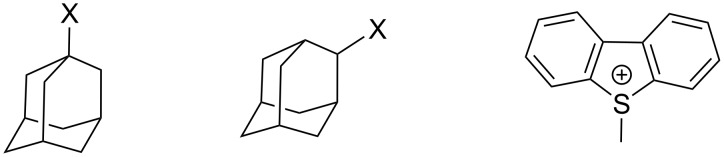
The chemical structures for the 1-adamantyl substrate, 2-adamantyl substrate, and the S-methyldibenzothiophenium ion (MeDBTh^+^). In the 1- and 2-substituted adamantyl substrates, X represents the anionic leaving group.

In the majority of solvolysis reactions, one also needs to evaluate the nucleophilic attack by the solvent which contributes modestly to the overall scheme for *tert*-butyl chloride solvolyses but appreciably for the solvolyses of methyl and primary alkyl halides. The original Grunwald–Winstein equation can be expanded to give a two-term Equation 2 [34,36–37], where *l* is the sensitivity to changes in *N*, which is a measure of solvent nucleophilicity. The original *N* scale was based on the specific rates of solvolysis of methyl tosylate [38] but, with this as the standard, there is a significant problem in assigning the required *m* value for use in the two-term equation. This problem was minimized by use of the *S*-methyldibenzothiophenium ion MeDBTh^+^ (as the trifluoromethanesulfonate) as the standard substrate [39–40] (Scheme 2). This solvolysis has as the leaving group a neutral molecule, dibenzothiophene, and it was shown from studies of 1-adamantyl derivatives [36] that ionic substrates of this type solvolyze in S_N_1 reactions with very little variation in specific rate with changes in solvent. The solvent nucleophilicity scale developed is termed *N*_T_ scale [36–37] and *N*_T_ = log(*k*/*k*_o_)_MeDBTh_^+^; where *k*_o_ is the value in 80% ethanol (20% water) and *k* is the value of the specific rate of solvolysis in another solvent of interest.


[2] $$\log(k/k_{o})=lN+mY$$

If a given solvolysis, such as of an alkyl chloride, is studied over a wide range of solvent types such as from ethanol (*N*_T_ = 0.37; *Y*_Cl_ = −2.52 to 97% HFIP (1,1,1,3,3,3-hexafluoro-2-propanol/3% H_2_O, by weight) with values of −5.26 for *N*_T_ and 5.17 for *Y*_Cl_, a discontinuity is sometimes observed in the plot. This discontinuity can usually be taken as evidence for a change in mechanism from bimolecular to unimolecular as is found, for example, in a study of the solvolyses of ethyl chloroformate or ethyl chlorothioformate, where there is a change in mechanism from bimolecular to unimolecular as one goes to more ionizing and less nucleophilic solvents [41]. In other instances, a good linear plot is obtained over a wide range of solvents, showing a strong tendency for either the unimolecular (low *l* value and high *m* value), as in the solvolyses of *tert*-butyl chloride [25,35], or a tendency to bimolecular solvolyses (high *l* value and intermediate *m* value, as is very well illustrated in a correlation of the rates of solvolyses of phenyl chloroformate [42].

When applying to the solvolyses of sulfonyl chlorides, with substitution resulting from attack at sulfur and not at carbon, there is another consideration involving the assumption that *N*_T_ and *Y*_Cl_ scales set up using substitution at carbon will also be applicable to substitution at sulfur. Those studies discussed in this review which involve the application of the extended Grunwald–Winstein equation should, through a consideration of goodness-of-fit parameters, such as correlation coefficient and *F*-test value, allow this question to be answered.

### 3. Studies by Bentley and co-workers

Koo, Bentley, Kang, and Lee [43] studied the solvolysis of 2,4,6-trimethylbenzenesulfonyl chloride in a mixture of water with acetone, acetonitrile, dioxane, ethanol or methanol in terms of rates of reaction and also product selectivities for the binary hydroxylic solvents, where both of the components (usually water plus an alcohol), can lead to products. Plots against *Y*_Cl_ values [35] were considered to give evidence for two reaction channels. Similar behavior was observed for the solvolysis of other electron-rich benzenesulfonyl chlorides [44] and for 2,4,6-triisopropylbenzenesulfonyl chloride [45], except that in the latter case the plot was against *Y* values [25,33]. Such an assignment needs to be treated with caution because the large contributions from solvent nucleophilicity effects to these solvolyses [16–17,26–27,30], even with electron-rich substrates, would be expected to lead to perturbations of plots against only solvent-ionizing power.

Shallow maxima observed when selectivity values (*S*, indicating the extent of product formation involving replacement of the chloride by the alkoxy group from an alcohol molecule reactive to replacement by the hydroxy group of a water molecule) could be due to two reaction channels varying in importance as the solvent composition is varied [43–45] but, as was shortly thereafter pointed out by Bentley and co-workers [46–48], the ratio will be influenced by a general base catalysis by a second alcohol or water molecule to the nucleophilic attack by the first alcohol or water molecule, leading to four possible reaction pathways, with two leading to replacement of the chloride by an alkoxy group and two to replacement by a hydroxy group.

In a 2007 article [49] with “Dual Reaction Channels” in both the title and the “Key Words,” there is extensive dispersal between the plots against *Y*_Cl_ for different aqueous organic solvents, with the 97% TFE point lying considerably below the other points, Arrhenius plots (3 temperatures) showed modest activation energies and very negative entropies of activation, consistent with an S_N_2 process. The S_N_2 process was given considerable support by the observation that an extended Grunwald–Winstein plot gave a very good correlation with all data points close to the linear plot. A maximum in selectivity values, consistent with a duality of mechanism was observed for aqueous ethanol but not for aqueous methanol. We will return to this system when, under a later subheading, we discuss applications of the extended Grunwald–Winstein equation [36–39].

Sulfonyl chlorides have relatively high heterolytic bond-dissociation energies [50] indicating that under the usual solvolytic conditions the formation of sulfonyl cations will be unfavorable. The mechanism is best considered as dominantly S_N_2, but possibly with some S_N_1 character, even in water or 97% TFE [51].

It has been proposed that, in addition to having an alcohol or water as the attacking nucleophile with a second alcohol of water as a general base, one can proceed in an alcoholysis reaction through a cyclic structure involving three molecules of the alcohol and the sulfur of the sulfonyl chloride present in the ring [52]. One would predict that such an extremely ordered pathway would have a very negative entropy of activation, but the required studies with temperature variation to investigate this aspect were not reported.

In reading the section “Substitutions at Sulphur” in the second edition of Ingold’s classical “Structure and Mechanism in Organic Chemistry” text [53], it is surprising to see that the section starts with the statement “No kinetic studies defining mechanisms of nucleophilic substitution at sulphur are known to the writer...”. Examination of this section of the text shows, however, that it is limited to a consideration of substitution at the sulfur of sulfinates and reactions of the general type illustrated in Equation 3 and reactions involving sulfonates are not considered.


[3]

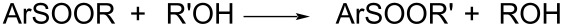



The hydrolyses in 1% dioxane/99% water have been studied at several temperatures by Houghton, Laird, and Spence for 27 variously substituted benzenesulfonyl chlorides [54]. They proposed that, consistent with previous reports, all reacted by a bimolecular nucleophilic substitution taking place at the sulfur.

### 4. Consideration of evidence from applications of the extended Grunwald–Winstein equation

Consistent with the bimolecular nature of the solvolyses of alkanesulfonyl chlorides and arenesulfonyl chlorides [49,55–67], poor correlations are obtained when the logarithmic rate data are correlated against only *Y*_Cl_ values, using Equation 1. When the *mY*_Cl_ term is joined by the *lN_T_* term (Equation 2), the application to sulfonyl chlorides varying from relatively simple to fairly complex structures (Table 1), and with use of solvents varying from aqueous acetone to aqueous fluoroalcohols, leads to acceptable to very good correlations, as assessed in terms of the accompanying correlation coefficients (*R*) and F-test values (*F*).

**Table 1:** Molecular structures of sulfonyl chlorides (ZSO_2_Cl) which have been studied kinetically in terms of the extended Grunwald–Winstein equation and/or in terms of kinetic solvent isotope effects (*k*_sie_ values) with relevant references.

molecular structure	references	molecular structure	references

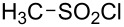 **1**	[59–60]	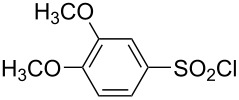 **10**	[49,62]
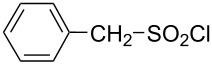 **2**	[62,68]	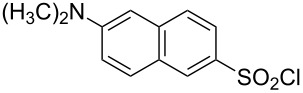 **11**	[65]
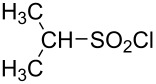 **3**	[58,61]	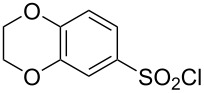 **12**	[66]
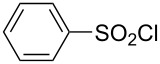 **4**	[57,63]	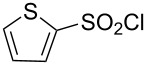 **13**	[56,62]
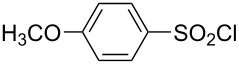 **5**	[62]	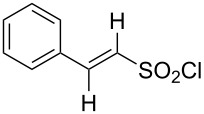 **14**	[63]
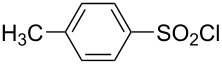 **6**	[62]	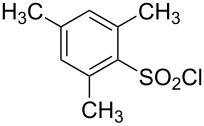 **15**	[43]
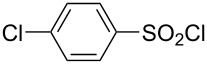 **7**	[69–70]	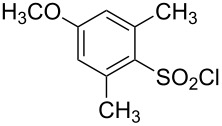 **16**	[44]
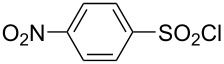 **8**	[63]	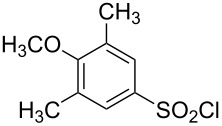 **17**	[71]
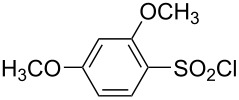 **9**	[64]	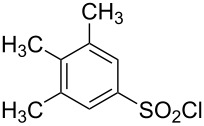 **18**	[71]

In Table 2 are assembled the calculated *l, m,* and *c* values (Equation 2), obtained from correlations in 19–43 solvents for thirteen sulfonyl chlorides (structures presented in Table 1), containing very simple to fairly complex substrates, which can be considered as aryl, arylalkyl, arylalkenyl, or heteroaryl sulfonyl chlorides. (**1** through **6** and **8** through **14** in Table 1 and these identifiers are used, and references [49,56–66,68]).

**Table 2:** Correlation of the specific rates of solvolytic displacement of chloride ions by nucleophilic interaction at the sulfur of sulfonyl chlorides (RSO_2_Cl) whose structures are tabulated and related to compound numbers in Table 1.

Compound^a^	*T,* °C^b^	*n* ^c^	*l* ^d^	*m* ^d^	*c* ^e^	*R* ^f^	*F* ^g^	*l*/*m*	Lit.^h^

**1**	45.0	39	1.17 ± 0.04	0.49 ± 0.02	0.23	0.981	454	2.39	[59–60]
**2**	45.0	29	0.87 ± 0.10	0.46 ± 0.06	0.09	0.874	42	1.89	[62,68]
	45.0	23^i^	0.80 ± 0.06	0.39 ± 0.04	0.21	0.947	95	2.05	[62]
**3**	45.0	19	1.28 ± 0.05	0.64 ± 0.03	0.18	0.988	333	2.00	[58,61]
**4**	35.0	29	1.26 ± 0.05	0.65 ± 0.03	0.13	0.979	304	1.94	[57,63]
**5**	25.0	38	1.07 ± 0.08	0.60 ± 0.03	0.22	0.967	254	1.78	[62]
**6**	25.0	34	1.19 ± 0.07	0.61 ± 0.02	0.20	0.975	305	1.95	[62]
**8**	35.0	21	1.52 ± 0.09	0.66 ± 0.05	0.10	0.968	134	2.30	[63]
**9**	25.0	30	0.93 ± 0.14	0.65 ± 0.06		0.918		1.43	[64]
**10**	25.0	40	1.24 ± 0.07	0.64 ± 0.03	0.14	0.967	264	1.94	[49,62]
**11**	35.0	31	0.96 ± 0.09	0.53 ± 0.03	0.10	0.955		1.81	[65]
**12**	25.0	28	1.00 ± 0.07	0.59 ± 0.03		0.948		1.69	[66]
**13**	25.0	34	1.35 ± 0.05	0.70 ± 0.02	0.28	0.983	455	1.93	[56,62]
**14**	45.0	43	1.24 ± 0.04	0.58 ± 0.02	0.07	0.982	542	2.14	[63]

^a^See Table 1 for structures of substrates. ^b^Temperature for the study. ^c^Number of data points. ^d^With associated standard error. ^e^With associated standard error of 0.04 to 0.06, except 0.09 for **8**. ^f^Correlation coefficient. ^g^*F*-test value. ^h^Publications from which the data points were assembled. ^i^Values for 2,2,2-trifluoroethanol/ethanol mixtures omitted.

It is found that, for the thirteen substrates, the *l*/*m* ratios vary only over a rather narrow range of 1.43 to 2.39. These values result from *l* and *m* values which are typical for S_N_2 substitutions The *l* values, sensitivities to changes in solvent nucleophilicity, are all close to unity (range of 0.80 to 1.52). This is to be expected because the prototype for S_N_2 solvolyses, which involves solvolyses at the methyl carbon of a methyl derivative is set at unity, for either the solvolyses of methyl *p*-toluenesulfonate [38] or of the *S*-methyldibenzothiophenium ion [39] when used as the standard S_N_2 substrate for solvolyses over a wide range of solvents and the range of *l* values suggests that the addition–elimination pathway appears to be disfavored for sulfonyl chlorides. The *m* values (Table 2) are consistent with the solvation electronics observed with the chloride leaving group in bimolecular processes [34].

**Figure 1:**
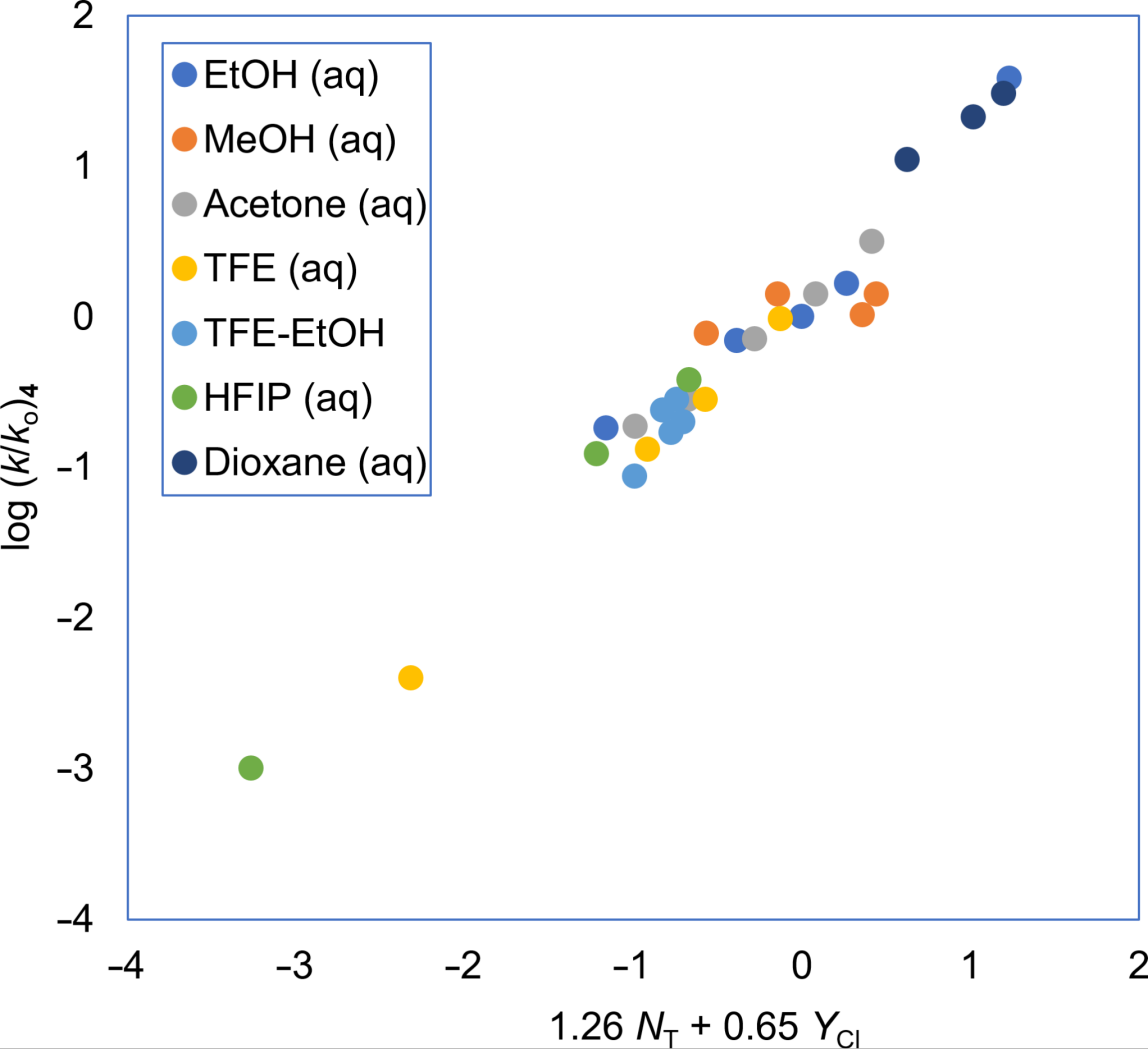
The S_N_2 reaction plot of log (*k*/*k*_o_) vs (1.26 *N*_T_ + 0.65 *Y*_Cl_) for the solvolyses of benzesulfonyl chloride (**4**) in pure and binary solvents at 35.0 °C. Figure 1 was redrawn using the data points from [63] (“Correlation of the Rates of Solvolysis of Two Arenesulfonyl Chlorides and of trans-β-Styrenesulfonyl Chloride – Precursors in the Development of New Pharmaceuticals“, © 2008 Z. H. Ryu et al., distributed under the terms of the Creative Commons Attribution 3.0 International License, https://creativecommons.org/licenses/by/3.0).

**Figure 2:**
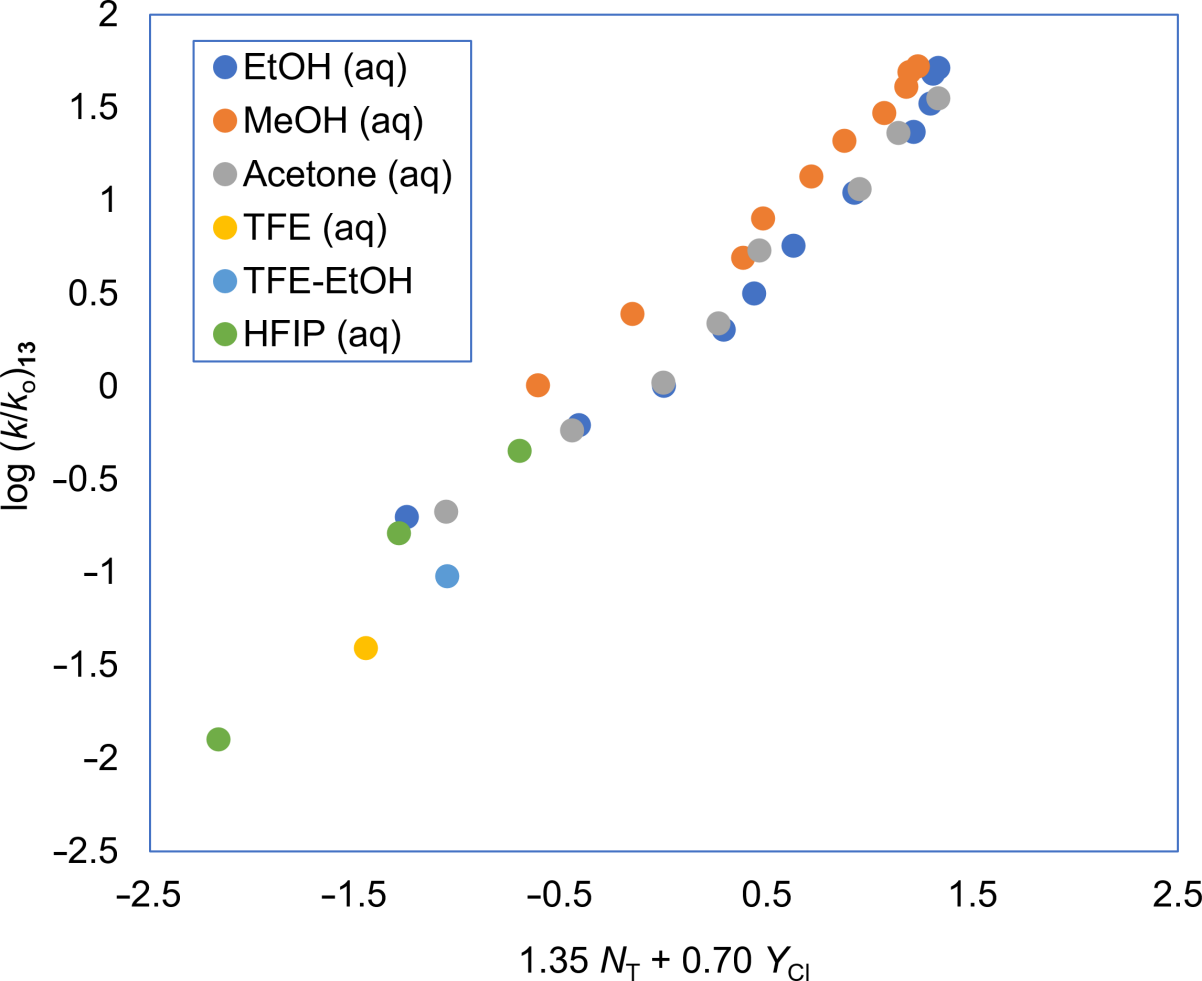
The S_N_2 reaction plot of log (*k*/*k*_o_) vs (1.35 *N*_T_ + 0.70 *Y*_Cl_) for the solvolyses of 2-thiophenesulfonyl chloride (**13**) in pure and binary solvents at 25.0 °C. The specific rate values are from [56] and [62].


For two of the systems studied, involving the substrates **4**, and **13** of Table 1, the application of Equation 2 is illustrated in Figure 1 and Figure 2. In both instances good linear plots are obtained. Substrate **2** is of considerable interest because, if the solvolysis is carried out in a deuterated solvent of type ROD in the presence of the conjugate base (OR^−^), it is found that there is deuterium uptake into the product. This is believed to be excellent evidence for the intermediacy of a sulfene formed by an elimination reaction promoted by the OR^−^ species, with the sulfene then rapidly adding a solvent molecule to give a final product which is identical to the direct substitution product, except that, with the deuterated solvent, deuterium uptake can easily be detected [72–74] (Equation 4). This reaction was found to occur only in the presence of reasonably high concentrations of the conjugate base of the alcohol solvent and it would not normally be observed under neutral solvolysis conditions.

[4]

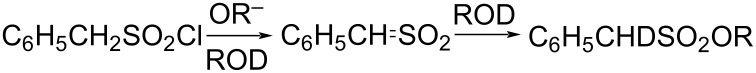



King has also studied the hydrolysis when the simplest structure for a tertiary alkyl group, the *tert*-butyl group, is attached to the sulfur of the sulfonyl group. Solvolysis of 2-methyl-2-propanesulfonyl chloride was found [75] to give evidence for the *tert*-butyl cation formation. It was proposed that the chlorosulfonyl group is about half as nucleofugic as a chloride ion (irrespective of whether it leaves intact on the SO_2_Cl^−^ ion or as SO_2_ + Cl^−^) (Equation 5).


[5]

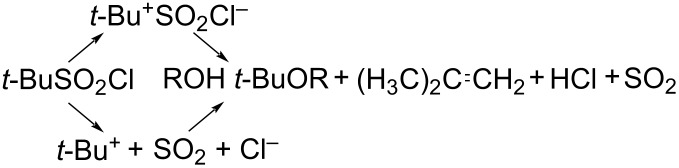



It would be of interest to extend this study to other RSO_2_Cl substrates, where the R forms a relatively stable carbocation, such as the 1-adamantanesulfonyl chloride and/or the diphenylmethanesulfonyl chloride [(C_6_H_5_)_2_CHSO_2_Cl], examples with intermediate cations which one would predict to be less stable and more stable, respectively, than the *tert*-butyl cation. These additional studies would be somewhat simplified in that an alkene, observed as a product from 2-methyl-2-propanesulfonyl chloride, would not be formed from the 1-adamantanesulfonyl or diphenylmethanesulfonyl chlorides.

While it is desirable to obtain information as regards the importance of solvent nucleophilicity as a rate-determining factor as above, from consideration in a sufficient number (twenty is a reasonable lower limit) of well-chosen (good variety as regards the combinations of *N* and *Y* values) solvents, this is a time-consuming process. If only an approximate value is required one can use the *k*_4OE_/*k*_97TFE_ ratio from measurements of the rates of solvolysis in only 40% EtOH (v/v) and 97% TFE (w/w) [25]. These solvents have virtually identical *Y*_Cl_ values of 2.75 and 2.83, respectively, but very different *N*_T_ values of −0.74 and −3.30, respectively [37], accordingly for these two mixed solvents, the logarithm of the ratio of the solvolysis rate for a given substrate in these two solvents (*k*_4OE_/*k*_97TFE_) will be governed almost entirely by 2.56 *l* (Equation 6).


[6] $$\log(k_{40\text{E}}/k_{97\text{TFE}})=2.56l$$

To take an example, for compound **14**, a value for *l* of 1.14 is obtained as opposed to a value of (1.24 ± 0.04) from the more rigorous extended Grunwald–Winstein equation treatment [63] (Table 2). Truncated comparisons of this general type had been earlier proposed by Cafferata and co-workers in a study of the solvolyses of alkyl fluorosulfates [76].

In discussing the *k*_4OE_/*k*_97TFE_ ratios, a good starting point is with the bridgehead-substituted 1-adamantyl chlorides, where a rear side S_N_2 process is impossible, with a value for the ratio of 0.83. The ratio raises to 1.38 for the tosylate but such variations are to be expected because different anionic nucleofuges do respond slight differently to solvent variation [25]. Inspection of Table 2 shows that the actual value for the ratio is sufficiently raised from unity for all the required substrates for which the required value in 97% TFE is available for them to be classified as bimolecular solvolyses. However, the values for the ratio do show a large variation, with a range from 74 for the 2,4-dimethylbenzenesulfonyl chloride (**8**) to 15000 for the *p*-nitrobenzenesulfonyl chloride (**7**). It is noteworthy that the ratio for the unsubstituted compound **4** of 2900 is reduced to 450 with a *p*-Me substituent (**5**) and further reduced to 300 with a *p*-MeO substituent (**6**), both electron-supplying substituents. It was reduced only slightly to 2430 in the presence of a *p*-Cl substituent and, as mentioned above, raised appreciably to 15000 in the presence of a powerfully electron-withdrawing *p*-NO_2_ group.

### 5. Evidence as regards the reaction mechanism from kinetic solvent isotope effect (*k*_sie_) measurements

Initial studies of solvent isotope effects in solvolysis reactions were largely carried out by comparing rates in H_2_O with those in D_2_O [75]. Low solubilities for many of the organic substrates studied [28] led to the adoption of studies in methanol and methanol-*d* as a favored alternative. It has been found, however, that for solvolysis of sulfonyl chlorides this factor is not as important as initially thought and solubilities in water are, for most (but not all) sulfonyl chlorides, usually sufficient for both *k*_H2O_/*k*_D2O_ and *k*_MeOH_/*k*_MeOD_ ratios to be attainable in the temperature range of 15–45 °C (Table 3).

**Table 3:** The specific rate ratios for solvolyses in 40% ethanol/60% H_2_O (v/v) relative to those in 97% TFE/3% H_2_O (w/w), *k*_40E_*/k*_97TFE_, and the kinetic solvent isotope effect (*k*_sie_) for solvolyses in methanol relative to solvolyses in methanol-*d* (*k*_MeOH_/*k*_MeOD_) and for solvolyses in H_2_O relative to solvolyses in D_2_O (*k*_H2O_/*k*_D2O_). The values for the ratios are at 25.0 °C unless otherwise stated.

Substrate^a^	*k*_40E_/*k*_97TFE_	Lit.	*k*_MeOH_/*k*_MeOD_	Lit.	*k*_H2O_/*k*_D2O_	Lit.

**1**	2010 (45 °C)	[59–60]	1.62; 1.51 (35 °C)	[63]	1.64 (18 °C); 1.57 (20 °C)	[28]
**2**	no value for 97% TFE		2.34 (35 °C)	[68]	very low solubility	
**3**	2790 (45 °C)	[63]	2.54; 2.41 (35 °C)	[61]	1.66; 1.45 (35 °C)	[61]
**4**	2900	[63]	1.79	[71]	1.56 (20 °C) 1.59 1.58 (15 °C)	[28] [71] [30]
**5**	300	[63]	1.58	[71]	1.37 1.41 (15 °C)	[71] [30]
**6**	450	[63]	1.72	[71]	1.49 1.50 (15 °C)	[71] [30]
**7**	2430	[69–70]	1.89	[71]	1.65	[71]
**8**	15000	[63]	2.31	[71]	1.76 1.82 (15 °C)	[71] [30]
**9**	74	[64]	1.74	[64]	1.86	[64]
**10**	386	[49]	1.45	[49]	1.35	[49]
**11**	no value for 97% TFE		1.88	[65]		
**12**			1.62	[66]		
**13**	no value for 97% TFE		2.34	[56]	1.47	[56]
**14**	85 (45 °C)	[63]	1.76 (45 °C)	[63]	1.46 (45 °C)	[63]
**15**	202	[43]				
**16**	89	[44]				
CF_3_SO_2_Cl	no value for 97% TFE		3.08 (45 °C)	[67]	2.24 (45 °C)	[67]
**17**			1.58	[71]	1.41	[71]
**18**			1.68	[71]	1.34	[71]

^a^The substrates corresponding to the substrate numbers are listed in Table 1.

Since the solvent isotope effects are quite small, ratios not far removed from unity in almost all cases, precise temperature control has to be coupled with a similarly precise method for obtaining the specific rates of solvolysis. When a strong acid is produced, as in Equation 7, conductivity measurements are usually the method of choice [31,77–78].


[7]

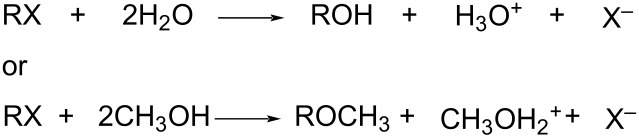



While very accurate measurements have been made, the ratios *k*_H2O_/*k*_D2O_ and *k*_MeOH_/*k*_MeOD_ have not proved to be very useful in assigning a structure to the transition state. One can in this regard quote Hammett from the second edition of his classical “Physical Organic Chemistry” text [79] “The hope, which at one time seemed bright, for a simple correlation of the solvent isotope effect with the mechanism of a reaction involving proton transfer or with the stoichiometric involvement of the solvent at the transition state has proved vain.”

Rossall and Robertson [30] found that, in the hydrolyses of *para*-substituted benzenesulfonyl chloride, the *k*_sie_ (*k*_H2O_/*k*_D2O_) increased in value as one goes from substrates with a *p*-MeO to the parent and on to the *p*-NO_2_-substituted substrate. Within Table 3, this corresponds to going from solvolyses of **5** to **6** to **4** to **7** and on to **8** (values of 1.37, 1.49, 1.59, 1.65, and 1.76 at 25.0 °C) and, for changes in *k*_MeOH_/*k*_MeOD_, values also increase steadily in the same sequence (values of 1.58, 1.72, 1.79, 1.89, and 2.31 at 25.0 °C) [71]. It was believed that these results, which differ dramatically from the very low sensitivity to structural change at a saturated carbon atom, reflect a more pronounced bondmaking by the solvents at the transition state for solvolyses of sulfonyl chlorides, involving attack at sulfur, than for attack at a saturated carbon atom. There is in Table 3 quite a number of values which have been reported on the basis of different investigations. It is noteworthy that there is uniformly a good agreement between these values and a firm foundation for attempts at an explanation. However, what Hammett pointed out [79] several years ago is still relevant today.

The section of the “Solvolysis Revisited” review [31] dealing with kinetic solvent isotope effects (pages 168–169) illustrates the extreme complexity of this topic and the uncertainty as to the extents to which the *k*_sie_ values are due to differences in initial states, due to differences in the chemical potentials of the transition state [80], or due to some other, as yet unidentified, source.

As a final topic, it is possible, indeed probable, that the complex associative structures in water and to a lesser extent in methanol lead to the simple two molecule formulation of the S_N_2 process for the hydrolysis being an oversimplification. The observation that the small molecule H_2_O is in bulk a liquid at room temperature requires a dynamic situation where water molecules are to a large extent associated by hydrogen-bonding to give dimers and larger aggregates. The S_N_2 process could therefore involve monomers, dimers and even larger aggregates as regards the attacking species. The dimers and larger aggregates can be considered as leading to general-base catalysis to an S_N_2 process. Alternatively, the process with attack by a dimer can be described as an S_N_3 process and, presumably, attack by a trimer would be S_N_4 and so forth. There is nothing wrong with either of these formulations provided the processes are suitably defined. Such a definition and explanation are given in Scheme 1 of the 2009 paper [69] by Bentley, Jones, Kang and Koo.

The present authors take into account the original Hughes and Ingold “Designation of Mechanism” [81]. “The molecularity of a reaction stage is defined as the number of molecules necessarily undergoing covalency changes,” (emphasis added), and for a composite reaction it is conveniently designated as “the molecularity of the rate-determining stage.” We personally favor the designation as bimolecular with or without general-base assistance from a second nucleophilic (basic) molecule.
